# Acrylated Chitosan Nanoparticles with Enhanced Mucoadhesion

**DOI:** 10.3390/polym10020106

**Published:** 2018-01-23

**Authors:** Shaked Eliyahu, Anat Aharon, Havazelet Bianco-Peled

**Affiliations:** 1 The Russell Berrie Nanotechnology Institute, Technion—Israel Institute of Technology, Haifa 3200003, Israel; shaked@campus.technion.ac.il; 2 Bruce Rappaport Faculty of Medicine, Technion—Israel Institute of Technology, Haifa 3200003, Israel; a_aharon@technion.ac.il; 3 Department of Hematology and Bone Marrow Transplantation, Rambam Health Care Campus, Haifa 3109601, Israel; 4 Department of Chemical Engineering, Technion—Israel Institute of Technology, Haifa 3200003, Israel

**Keywords:** chitosan, acrylated chitosan, nanoparticles, mucoadhesion, mucosal membranes, mucoadhesive polymers, retention

## Abstract

The aim of this study was to investigate the effect of acrylate modification on the mucoadhesion of chitosan at the nanoscale. Nanoparticles were fabricated from acrylated chitosan (ACS) via ionic gelation with tripolyphosphate and were characterized in terms of size, zeta potential, stability, and nanoparticle yield. Chitosan (CS) nanoparticles, serving as a control, were fabricated using the same procedure. The mucoadhesion of the nanoparticles was evaluated using the flow-through method after different incubation periods. The retention percentages of ACS nanoparticles were found to be significantly higher than those of CS nanoparticles, for all studied time intervals. An additional indication for the increased mucoadhesion of ACS nanoparticles was the increase in particle size obtained from the mucin particle method, in which mucin and nanoparticles are mixed at different ratios. NMR data verified the presence of free acrylate groups on the ACS nanoparticles. Thus, the improved mucoadhesion could be due to a Michael-type addition reaction between the nanoparticles and thiol groups present in mucin glycoprotein, in addition to entanglements and hydrogen bonding. Overall, ACS nanoparticles exhibit enhanced mucoadhesion properties as compared to CS nanoparticles and could be used as vehicles for drug delivery systems.

## 1. Introduction

The ability to adhere to mucosal surfaces, termed mucoadhesion, has attracted much attention in the last few decades. This adhesive property is considered valuable for pharmaceutical purposes, as mucosal drug delivery has great potential to provide improved drug absorption and bioavailability [1]. Drug residence time on mucosal surfaces can be prolonged using polymers designed to attach to mucosal membranes. Such mucoadhesive dosage forms are a useful tool for mucosal drug delivery. Polymeric nanoparticulate mucoadhesive carriers have even greater potential. Not only can they adhere to mucosal tissues, but they also offer increased surface area and enhanced bioavailability by protecting the drug from degradation [2].

Chitosan (CS) is a semisynthetic biocompatible polysaccharide derived from the deacetylation of the acetyl group in chitin [3]. CS has emerged as a promising vehicle for drug delivery, not only because of its mucoadhesion, but also because it can transiently open the tight junctions of epithelial tissues, increasing paracellular transport of drugs [4]. The mucoadhesive properties of CS arise from its ability to establish electrostatic interactions, hydrogen bonding, and hydrophobic interactions with mucin glycoprotein [5]. CS can be chemically or physically crosslinked, creating particles at the micro- or nanoscale [6,7]. The inherent mucoadhesion properties of CS nanoparticles can be further enhanced by thiolation, more specifically by grafting thiol-containing side groups to CS. Thiolated CS nanoparticles are capable of forming disulfide bridges with mucin’s cysteine residues; as a result they demonstrate enhanced adhesion [8,9,10,11,12]. For example, the mucoadhesive force of nanoparticles composed of chitosan-4-thiobutylamidine was shown to be more than two-fold greater than that of CS nanoparticles [10].

Our group has pioneered the concept of acrylated polymers: hydrophilic macromolecules carrying acrylate end groups that covalently bind to mucin’s thiol via a Michael-type addition reaction, which can take place in a physiological environment. We have established that acrylation of polymers, including polyethylene glycol (PEG) [13], alginate [14], poly(ethylene oxide)/poly (propylene oxide) blockcopolymer (Pluronic^®^127) [15], and chitosan [16], improves their mucoadhesive properties in comparison to the non-modified polymers. Previous studies have also demonstrated that acrylation does not cause any cytotoxic effect in neonatal human foreskin fibroblasts [14]. In particular, acrylated chitosan (ACS) was shown to be more mucoadhesive than both chitosan (CS) and thiolated chitosan [16]. The ability of ACS to form nanometric particles has not been explored before. Furthermore, it was not known whether the superior mucoadhesion properties of acrylated polymers are preserved at the nanoscale.

CS nanoparticles are commonly fabricated by ionic gelation using the multivalent ion tripolyphosphate (TPP) [17]. The formed nanoparticles are biodegradable, mucoadhesive, and nontoxic [18,19]. Even though methods for the formation of CS nanoparticles are well established, it is not straightforward to assume that TPP will react with modified CS in the same manner as with CS. Chemical modifications of CS typically consume its positively charged groups, hence affecting the ionic interactions between CS and TPP. In the specific case of acrylation, reducing electrostatic interaction could be accompanied by steric hindrance induced by the grafting of bulky PEG chains.

The current research had two main objectives. The first was to evaluate whether TPP can be utilized for fabrication of acrylated CS nanoparticles and investigate the conditions for their formation. The second was to investigate the influence of the chemical modification on the mucoadhesion of this nanometric system. Acrylated CS was previously shown to have superior mucoadhesive properties [16]. However, a recent review by das Neves et al. emphasized the possibility that bulk polymers will exhibit different behaviors to nanoscale particles [2]. 

Here, we report for the first time on the formation and characterization of acrylated CS nanoparticles. A procedure for the fabrication of ACS nanoparticles was developed, and the resulting nanoparticles were characterized in terms of size, stability, and particle yield. The mucoadhesive properties were studied with respect to the polymer-to-mucin ratio, by measuring the mucoadhesive index [20]. In addition, the flow-through method was used to measure the retention percentage of the nanoparticles on porcine intestine. The retention was examined with respect to the time of incubation with the mucosal tissue. CS nanoparticles served as a control in all measurements. We demonstrate that ACS nanoparticles exhibit enhanced mucoadhesion properties compared to CS nanoparticles and could be used as vehicles for drug delivery systems.

## 2. Materials and Methods 

### 2.1. Materials

Low molecular mass chitosan (Mw 207 kDa, deacetylation degree 77.6%), mucin type II from porcine stomach, l-α-phosphatidylcholine, fluorescein isocyanate, and fluorescamine were obtained from Sigma Aldrich (Rehovot, Israel). Sodium tripolyphosphate was purchased from Alfa Aesar (Lancashire, UK). Sodium chloride, dichloromethane, and NaOH were obtained from Bio-Lab Ltd. (Jerusalem, Israel). Sodium taurocholate was purchased from Carbosynth (Berkshire, UK) and maleic acid from Arcos Organics (Geel, Belgium). Acetic acid glacial and dimethyl sulfoxide (DMSO) were purchased from Merck (Darmstadt, Germany). Polyethylene glycol diacrylate (PEGDA) with Mw of 10 kDa was obtained from the laboratory of biomaterials and regenerative medicine at the Department of Biomedical Engineering, Technion, Israel. Fresh porcine small intestine was obtained from the Pre-Clinical Research Authority, Technion, Israel. All research protocols involving live animals are subjected to the approval of the institute’s ethical committee for animal experiments (IACUC). All procedures comply with the Animal Welfare Act of 1966 (P.L. 89–544), as amended by the Animal Welfare Act of 1970 (P.L. 91–579) and 1976 (P.L. 94–279). The protocol was approved by the Ethics Committee of the Technion (approval # IL-012-02-2016). The intestines were harvested immediately after the animals were euthanized. The porcine intestine was sliced shortly after removing it from the animal, and stored at −20 °C until further use. 

### 2.2. Synthesis of Acrylated Chitosan

Acrylated chitosan (Figure S1) was synthesized as described by Shitrit and Bianco-Peled [16]. We give a brief description of the process here. One g CS was dissolved in 100 mL 2 *v*/*v* % acetic acid at room temperature and stirred overnight. Then, 1 g PEGDA was added and stirred for 15 min. The reaction mixture was incubated for 3 h in the dark under shaking at the speed of 100 rpm at 60 °C. Next, the reaction mixture was dialyzed in the dark against 5 L of double distilled water (DDW) for 3 days. After dialysis, the product was filtered with a Buchner funnel, lyophilized at 0.01 mbar and −30 °C, and stored at −20 °C until further use.

### 2.3. Synthesis of Fluorescein Isocyanate (FITC)-Labeled Polymers

Fluorescently labeled CS and ACS were synthesized by reacting the primary amino group of CS and the isothiocyanate group of fluorescein according to the procedure used by Tallury et al. [21] with minor modifications. One g of CS was dissolved in 100 mL of 2 *v*/*v* % acetic acid and stirred overnight. Fluorescein isocyanate (FITC) solution was prepared by dissolving 25 mg FITC in 100 mL methanol and adding it to the CS solution. The reaction was allowed to proceed under magnetic stirring in the dark at room temperature overnight. Next, 0.1 M NaOH was added, resulting in precipitation of the labeled CS that was then separated from the solution by filtration with a Buchner funnel. The product was washed with a mixture of ethanol/water (70:30) until no fluorescence was detected in the washing solution. The labeled CS was dissolved in 100 mL of 1 *v*/*v* % acetic acid and dialyzed against 5 L of the same solvent, followed by dialysis against DDW for 48 h. The final product was lyophilized at 0.01 mbar and −30 °C and stored at −20 °C until further use. 

To prepare ACS-FITC, 500 mg of lyophilized CS-FITC was dissolved in 2 *v*/*v* % acetic acid. Then, 500 mg of PEGDA was added to the labeled CS solution under continuous stirring for 15 min. The reaction was allowed to proceed at 60 °C under shaking for 3 h. The labeled ACS was dialyzed in the dark against 5 L DDW for 48 h. Finally, the labeled ACS was filtered, lyophilized at 0.01 mbar and −30 °C, and stored at −20 °C until further use.

### 2.4. Polymer Characterization

#### 2.4.1. Nuclear Magnetic Resonance (NMR)

CS and ACS were dissolved in a mixture of 2 *v*/*v* % CD_3_COOD and 98 *v*/*v* % D_2_O and studied using a 400 MHz Bruker ARX400 (Bruker, Billerica, MA, USA) at ambient temperature. Lyophilized CS and ACS nanoparticles were redispersed in the same solvent prior to the NMR measurements.

#### 2.4.2. Fluorescamine Test for Quantitative Determination of Acrylate Groups

To determine the acrylation percentage of ACS, we modified the method proposed by Shitrit and Bianco-Peled [16] by replacing ninhydrin with fluorescamine assay [22]. A series of CS or ACS solutions with decreasing concentrations was prepared using 2 *v*/*v* % acetic acid as a solvent. Samples of 140 µL of 0.1 M boric acid solution at pH 8 were placed in a black 96-well plate, and 10 µL of the polymer solution was added to each well. Then 50 µL of 1 mg/mL fluorescamine solution in DMSO was added to each well and mixed in the dark under shaking at 125 rpm for 10 min at room temperature. The fluorescence was measured using a UV–vis BioTek Synergy HT plate reader (BioTek Instruments, Winooski, VT, USA) with an excitation wave length of 360 ± 40 nm and an emission wave length of 460 ± 40 nm. Background fluorescence was determined using a solution of 2 *v*/*v* % acetic acid. The obtained fluorescence was plotted against the polymer concentration. The slope was used to calculate the acrylation percentage as follows:

(1) $$Acrylation\;percentage=100\%\cdot \left(1-\frac{a_{pol}}{a_{CS}}\right),$$

where *a_pol_* and *a_CS_* are the slopes obtained from the *a_CS_* curve and the CS curve, respectively.

### 2.5. Fabrication of Nanoparticles 

CS and ACS nanoparticles were fabricated using the ionic gelation technique, where the positively charged amine residues of CS form electrostatic forces with the multivalent anion TPP [23]. A polymer to crosslinker ratio of 8:1 was chosen following preliminary experiments that yielded comparable particle size for both CS and ACS nanoparticles.

CS (0.1 *w*/*v* %) or ACS (0.2 *w*/*v* %) was dissolved in 0.1 *v*/*v* % acetic acid containing 0.1 M NaCl and stirred for 20 h. The mole excess of acetic acid, with respect to the amine groups of CS, is 300% for CS solutions and 2200% for ACS solutions. The pH was adjusted to 4, since at this value TPP possesses three negative charges that can physically crosslink the chitosan [24]. The concentration of NaCl was set to 0.1 M according to Huang and Lapitsky [25] and Antoniou et al. [26] to maintain stable particle size and low particle size distribution. A volume of 0.833 mL of TPP (0.025 *w*/*v* % in 0.1 M NaCl) aqueous solution was added dropwise to 1.667 mL of CS solution under magnetic stirring. Similarly, a volume of 0.5 mL of TPP (0.1 *w*/*v* % in 0.1 M NaCl) aqueous solution was added dropwise to 2 mL of ACS solution under magnetic stirring. The solutions were stirred for an additional 15 min at room temperature. FITC-labeled nanoparticles were created according to the same procedure using labeled polymers.

### 2.6. Nanoparticle Characterization 

#### 2.6.1. Size and Zeta Potential

Dynamic light scattering (DLS) was used to determine the average size, size distribution, and zeta potential of the nanoparticles. Measurements were performed in triplicate using a Zetasizer Nano ZSP (Malvern Instruments Ltd., Malvern, UK). A volume of 500 µL from the sample was placed in a polystyrene cuvette and measured at 25 °C. 

#### 2.6.2. Yield

A volume of 50 mL of nanoparticle solution was prepared in triplicate and the pH was adjusted to 5.9 using 5 M NaOH. The solution was centrifuged in a Heraeus Multifuge X3R centrifuge (ThermoFisher Scientific, Waltham, MA, USA) at 4 °C and 18,000 g for 1 h. Cycles of slow freezing at −20 °C and thawing at room temperature were performed in order to destabilize the nanoparticles and cause their aggregation, hence facilitating the precipitation. Nanoparticle pellets were lyophilized and weighed. The yield was calculated as follows:

(2) $$Yield=\frac{M_{NPs}}{M_{T}}\times 100\%$$

where *M_NPs_* is the mass of CS-TPP nanoparticles recovered and *M_T_* is the combined total weight of CS and TPP used in the formulation. 

### 2.7. Mucoadhesion Studies

#### 2.7.1. Nanoparticle Retention Studies 

Retention studies using porcine intestine as a substrate were performed as previously described by Eshel-Green et al. [27]. Portions of porcine intestine tissue (1.5 × 3 cm) were thawed for 5 min at 100% humidity and 37 °C. FITC-labeled nanoparticles in a volume of 50 µL (0.00135 mM) were placed on the mucosal side of the tissue and incubated at 37 °C and 100% humidity for different times in the dark. Then, the tissue was set in a trough made of half a pipe, placed at a 45 degree angle in relation to the table. Using a syringe pump, a flow of buffer simulating the fasted state condition in the upper small intestine (FaSSIF-V2, prepared according to [28]), was dripped onto the substrate at a constant rate of 2 mL/min. Small aliquots of 200 µL each were collected continuously and analyzed fluorescently using a UV–vis spectrophotometer (BioTek Instruments, Winooski, VT, USA) with excitation wave length of 485 ± 20 nm and emission wave length of 525 ± 20 nm. The aliquots were analyzed for their nanoparticle concentration using a calibration curve of the labeled nanoparticles in FaSSIF-V2 buffer. All measurements were performed in triplicate. At the end of the experiment, the tissues were observed under a Nikon Eclipse TS100 fluorescence microscope equipped with a Nikon digital sight DS-Fi2 camera. 

#### 2.7.2. Nanoparticle-Mucin Aggregation 

The mucoadhesion of the nanoparticles was examined using the mucin solution method described by Raskin et al. [20], with minor modifications. First, a soluble fraction of mucin was obtained. A solution of 5 mg/mL was prepared by hydrating powdered mucin in DDW and employing magnetic stirring for 3 h at room temperature. The insoluble fraction was removed by centrifugation at 4000 g for 10 min. The supernatant was collected, lyophilized, and stored at 4 °C until further use. The lyophilized mucin was dissolved in DDW at predetermined mucin ratios *f*, defined as:

(3) $$f=\frac{w_{mucin}}{w_{mucin}+w_{NPs}}$$

where *w_mucin_* is the mass of mucin and *w_NPs_* is the nanoparticle mass.

Solutions of soluble mucin were pre-incubated at 37 °C. Freshly prepared nanoparticle solutions were size measured by DLS before the experiment. The aggregation with mucin solutions was determined by adding 150 µL of nanoparticle solution to 1 mL mucin solution under magnetic stirring at 37 °C for 1 min. The final concentration of the nanoparticles in the solution was 0.0087 *w*/*v* %. The final size was measured by DLS immediately after the time noted. The mucoadhesive index, indicating the increase in particle size, was calculated according to:

(4) $$MI=\frac{d}{d_{0}}$$

where *d*_0_ and *d* denote the diameter of the particle before and after incubation with mucin, respectively. Measuring the size of nanoparticles mixed with a 1 mL of DDW served as a negative control. All measurements were performed in triplicate. 

### 2.8. Statistical Data Analysis 

Microsoft^®^ Excel software was used for the statistical analysis of the data. Standard errors of the mean (SEM) were calculated and presented for each treatment group. Statistical analysis was performed using the one-way analysis of variance (ANOVA) for comparisons between multiple treatments, while the one-tailed Student’s *t*-test was used for comparison between two treatments. A *p*-value of 0.05 was considered statistically significant.

## 3. Results and Discussion

### 3.1. Acrylation of Chitosan 

Chitosan was covalently modified by grafting PEGDA with a molecular mass of 10 kDa. This molecular mass was selected following a previous study that demonstrated better mucoadhesion properties of CS modified with 10 kDa PEGDA compared to CS modified with 0.7 kDa PEGDA [16]. After the synthesis, the product was characterized using NMR spectra, and the presence of free acrylate end groups was verified (Figure S2). In order to quantify the acrylation percentage of the products, we modified the method developed by Shitrit and Bianco-Peled [16]. The ninhydrin reagent was replaced with fluorescamine, making the procedure easy to execute at room temperature with only one stage of mixing. Fluorescamine binds to primary amine groups, resulting in fluorescence that is proportional to the amine concentration. Simultaneously, the excess reagent undergoes hydrolysis [22]. To validate the method, the acrylation percentage was also determined using the ninhydrin assay and found to be identical for both methods. This percentage was calculated from Equation (1) and found to be 65%. The molecular mass of ACS was calculated from the acrylation percentage and was found to be 5.9 × 10^6^ Da. The PEGDA grafting percentage is less than 100%, indicating that there are amine groups which are free to bind TPP to produce ionically crosslinked nanoparticles. 

It is noted that grafting hydrophilic PEG chains improved the solubility of ACS in aqueous medium. Contrary to CS, which is soluble only in acidic solutions, ACS was found to be soluble also in neutral pH value.

### 3.2. Fabrication of Nanoparticles

**Figure 1:**
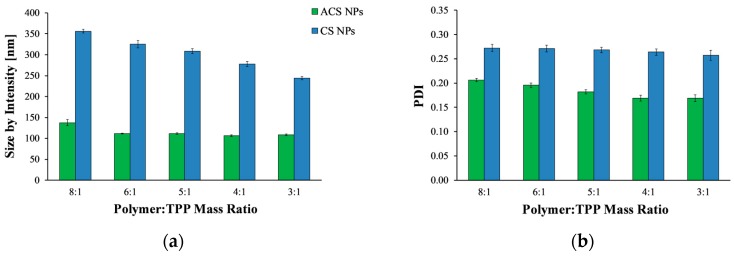
(**a**) Mean hydrodynamic diameter and (**b**) polydispersity index (PDI) value of acrylated chitosan (ACS) (green) and chitosan (CS) (blue) nanoparticles prepared at different polymer to crosslinker mass ratios.

Polymeric nanoparticles were prepared using the ionic gelation process with TPP, wherein the positively charged amine residues in CS or ACS form electrostatic forces with the multivalent anion TPP. To allow better comparison between CS and ACS, we attempted to formulate nanoparticles similar in size by screening several possible experimental variables. CS and ACS nanoparticles crosslinked with TPP were fabricated using different polymer to crosslinker mass ratios and a constant polymer concentration of 2 mg/mL, and their average size and polydispersity index (PDI) were then determined (Figure 1). Overall, the size of CS nanoparticles decreases significantly with an increase in polymer to crosslinker ratio (ANOVA, *p* < 0.0001), in agreement with previously published results [7,29,30]. The average diameter of CS nanoparticles fabricated using the lowest polymer to crosslinker mass ratio of 8:1 was 356.0 ± 4.5 nm, while at the highest mass ratio of 3:1 the average diameter was reduced to 244.2 ± 4.2 nm. The reason for this decrease in size was found to be the increase in particle compactness [7].

Figure 1a shows that, in contrast to CS nanoparticles, the size of ACS nanoparticles did not change significantly with an increase of polymer to crosslinker ratio from 6:1 to 3:1 (ANOVA, *p* > 0.05). Only at a polymer to crosslinker ratio of 8:1 was the diameter significantly larger than it was at higher ratios (*p* < 0.05). This relatively minor influence of polymer to crosslinker ratio on ACS particle size may be attributed to the fact that 65% of the amine groups on the ACS backbone are conjugated to PEG, whereas only 35% are free to react with TPP. Therefore, fewer amine groups participate in the crosslinking. The ionic gelation process is mechanistically similar to the precipitation of colloids [25]. When TPP is added dropwise to a CS solution, every drop forms a nucleus, which is the source of the aggregation of polymer chains, forming a primary small nanoparticle. Primary nanoparticles aggregate into larger secondary colloids by bridges of TPP ions [31]. The balance between three processes—primary aggregation, secondary aggregation, and aggregate breakage due to stirring—determines the particle size. The grafting of acrylate groups on the CS backbone might weaken or interrupt the interactions of polymer chains via TPP ions. Consequently, the number of coagulation events (i.e., secondary aggregation) decreases, as does the rate of particle formation. Overall, the balance is tilted towards smaller particles, as secondary aggregation occurs less frequently, while TPP drops continue to form new particles. 

Figure 1b displays the PDI values measured by DLS. PDI is a parameter calculated from a cumulants analysis of the DLS measured intensity autocorrelation function [32]. Low PDI values indicate homogeneous distribution, while high PDI points to polydispersity [26,33]. All nanoparticle formulations exhibit relatively low (mid-range) PDI values, over which the distribution algorithms best operate. For both CS and ACS nanoparticle formulations, as the polymer to crosslinker ratio increases, the PDI value decreases (ANOVA, *p* < 0.0001, *p* < 0.0005 respectively), indicating a narrow size distribution.

As evident from Figure 1, none of the tested polymer to crosslinker ratios could be used to obtain CS and ACS nanoparticles of similar size. Furthermore, many attempts to alter the size of ACS nanoparticles by varying the polymer and salt concentration were not successful since they all led to the formation of nanoparticles of about the same size (data not shown). Considering these results, we attempted to change the composition of the solutions used to create CS nanoparticles. This time, the polymer to crosslinker mass ratio was kept constant at 8:1, and the nanoparticles were created using different initial concentrations of polymer and crosslinker. DLS measurements were performed to determine the nanoparticle size (Table 1). Decreasing the initial TPP concentration from 1 mg/mL to 0.5 and 0.25 mg/mL resulted in smaller particles. A further decrease in the initial CS concentration from 2 mg/mL to 1 mg/mL led to the formation of particles of about 200 nm, a size comparable to that of the ACS nanoparticles. Therefore, CS nanoparticles for further study were prepared using 1 mg/mL of CS and 0.25 mg/mL of TPP at an 8:1 mass ratio of polymer to crosslinker.

### 3.3. Stability of the Nanoparticles

**Figure 2:**
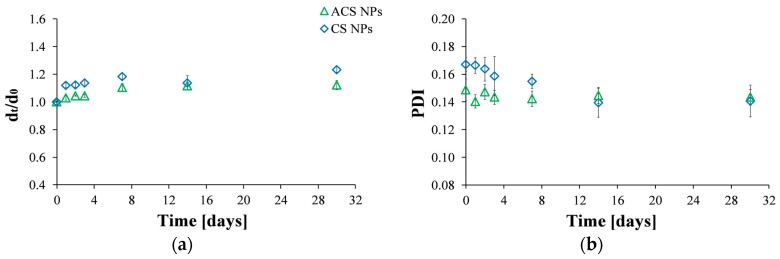
Size stability over time measured by: (**a**) Hydrodynamic diameter by intensity at time t divided by the initial diameter at time zero; (**b**) PDI values of ACS (green triangle) and CS (blue diamond) nanoparticles fabricated at an 8:1 mass ratio of polymer to crosslinker.

Particles are considered stable if their zeta potential value is between +30 mV and −30 mV [34]. The zeta potential of CS nanoparticles was found to be 27.7 ± 0.3 mV, while the zeta potential value obtained for ACS nanoparticles was 13.7 ± 0.2 mV. Since these values may indicate that the stability of the nanoparticles is less than optimal, we investigated particle stability by following the particle size and the PDI values as a function of time for a period of one month (Figure 2). The diameter of the nanoparticles, normalized to the initial size at the time of preparation, indicates that CS and ACS nanoparticle formulations display a similar pattern: the diameter slightly increases shortly after preparation with no further size changes after seven days (Figure 2a). The diameter of CS nanoparticles increased by 23% while that of ACS nanoparticles displayed a smaller increase of 12% over a long period of 30 days. Figure 2b displays the PDI values measured by DLS. As was mentioned before, low PDI values indicate homogeneous distribution, while high PDI points to polydispersity [26,33]. The formulations of the two nanoparticle types exhibit relatively low (mid-range) PDI values, over which the distribution algorithms best operate. The PDI of the CS nanoparticles gradually decreased during the first 14 days after preparation, with no further changes after this time. In contrast, the PDI of the ACS nanoparticles remained constant throughout the experiment. The smaller diameter change and constant PDI of the ACS nanoparticles support our hypothesis that they are more stable than CS nanoparticles. ACS nanoparticles possess bulky PEGDA chains that keep them from aggregating and maintain their stability, while primary CS nanoparticles aggregate into larger secondary colloids by bridges of TPP ions.

### 3.4. Nanoparticle Yield

To further investigate the differences between ACS and CS nanoparticle formulations, we determined the nanoparticle yield. Yield can be characterized by separating the nanoparticles with centrifugation, followed by either quantification of chitosan content in the supernatant or determining the weight of precipitated chitosan nanoparticles [35,36]. The downside of these approaches is that some of the smaller nanoparticles cannot undergo precipitation by centrifugation alone due to their light weight. A recent study by Rampino et al. showed that adding TPP to a CS nanoparticle suspension increases the amount of precipitated nanoparticles due to their flocculation, yet the final yield was still low, indicating that not all the nanoparticles precipitated [36]. Furthermore, if free chitosan chains coexist with the nanoparticles, TPP addition might nucleate new nanoparticles, hence increasing the measured yield to levels higher than the true value. 

Here we suggest an improved method to measure the nanoparticle yield. Instead of centrifugation alone, or adding TPP prior to centrifugation, we conducted several cycles of freezing and thawing prior to centrifugation. This method is commonly used to assess solid content following emulsion polymerization. Slow freezing causes the formation of ice crystals, which force the particles to be in close proximity in a small volume. As a result, the particles aggregate and stay in this form even when they are re-thawed [37,38]. 

**Figure 3:**
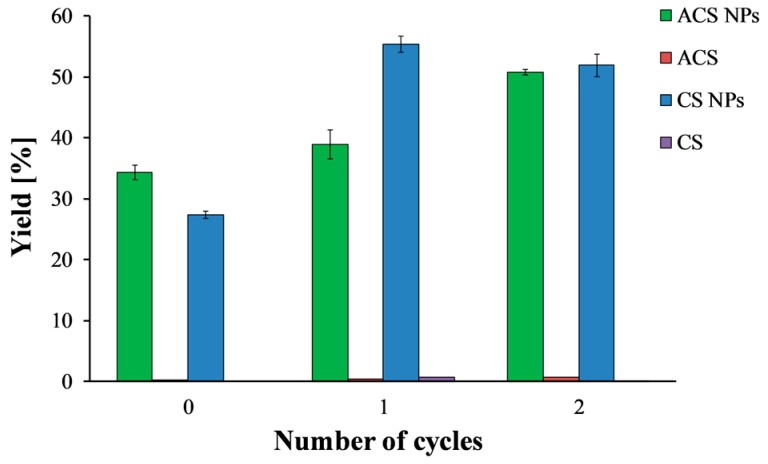
ACS (green) and CS (blue) nanoparticle yield as a function of the number of freeze/thaw cycles. ACS (purple) and CS (red) polymer solutions were examined as a control.

Figure 3 shows the precipitated mass after lyophilization, normalized to the total solid mass of polymer and crosslinker, as a function of the number of freeze/thaw cycles. Solutions of CS and ACS that were subjected to the same treatments—i.e., freeze/thaw cycles and centrifugation—were included in the study as controls. After one freeze/thaw cycle, the nanoparticle yield increased from 27.4 ± 0.01% to 55.4 ± 0.02% for CS and from 34.3 ± 0.02% to 39.0 ± 0.04% for ACS. These findings support the suggestion that some of the smallest nanoparticles cannot be separated by centrifugation alone and only disruption of their stability by freezing and thawing allows precipitation. Thus, after centrifugation, there is an increase in the precipitated nanoparticle mass. Adding another freeze/thaw cycle did not affect the CS nanoparticle yield, but it increased the ACS nanoparticle yield to 50.8 ± 0.01%.

It is important to note that one or two cycles of freezing and thawing did not affect the solubility of the polymer solutions and resulted in a negligible precipitate. After three cycles of freezing and thawing, a sudden increase in the precipitate amount was observed for both nanoparticle formulations (data not shown). However, the amount of precipitate in the control experiment increased considerably as well, indicating an undesired effect of precipitation of free polymer chains. Therefore, the maximum yield is 55.4 ± 0.02% for CS nanoparticles and 50.8 ± 0.01% for ACS nanoparticles. These results revealed that CS and ACS nanoparticles with the same polymer to crosslinker ratio but different initial concentrations resulted in comparable nanoparticle yield. These yield values agree with the ones reported by Antoniou et al., who found a yield of 45% for CS-TPP nanoparticles prepared using a polymer to crosslinker ratio of 9:1 and CS concentration of 0.5 mg/mL [26].

### 3.5. Mucoadhesion Characterization

Many methods have been suggested to characterize mucoadhesion of nanoparticles [1]. Some of these methods are considered to give a direct measure of mucoadhesion as they assess the interactions of the nanoparticles with a mucus covered tissue. Others provide non-direct evidence for mucoadhesion by quantifying the interactions between mucins and nanoparticles. Out of these methods we chose flow-through as a representing direct method and nanoparticle-mucin aggregation as a representative non-direct method. The flow-through method allowed us to investigate for the first time the effect of incubation time on mucoadhesion. The nanoparticle-mucin aggregation method was used here to examine the influence of various mucin-polymer ratios. 

#### 3.5.1. Nanoparticle Retention on Mucosal Surfaces

**Figure 4:**
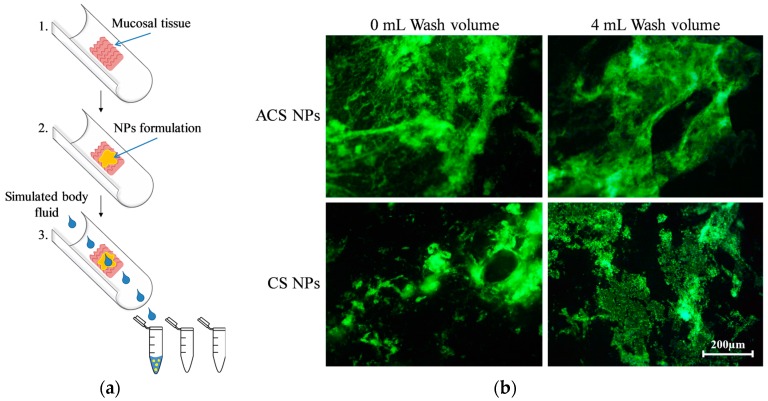
(**a**) Schematic representation of the flow-through method; (**b**) Fluorescent microphotographs of porcine intestine tissue samples with fluorescein isocyanate (FITC)-labeled ACS nanoparticles and CS nanoparticles, washed off with simulated intestinal fluid at a flow rate of 2 mL/min after incubation of 10 min.

The flow-through method was developed by Rao et al. to examine the mucoadhesion of polymers and coated microparticles [39]. Later, this technique was used to assess the adhesion of micro- and nanoparticles [40,41]. To quantify the ability to retain on the mucosa, a fluorescently labeled formulation is placed on a mucosal membrane. A flow of buffer, simulating the pH and salt content in the body, washes the polymer formulation off the tissue at a constant rate (Figure 4a). The mucosal membrane can be imaged for the remaining fluorescence [41], and the elution buffer can be analyzed to determine the polymer concentration washed away from the mucosa [27].

In the current study, we used this technique to evaluate and compare the retention of the two nanoparticle formulations on porcine small intestine. Fluorescent images were taken before and immediately after washing with 4 mL buffer, verifying the retention of FITC-labeled nanoparticles (green fluorescence) for both CS and ACS formulations after washing (Figure 4b). The dark fields in the images can be related to microscopic areas in the mucosal tissue with different affinity for the labeled particles. 

**Figure 5:**
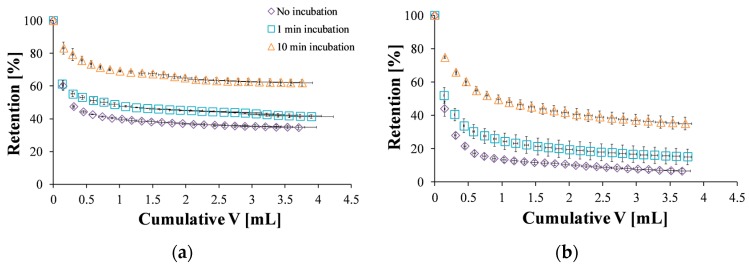
Retention profile of: (**a**) ACS nanoparticles without incubation (purple diamond), one minute of incubation (blue square), and ten minutes of incubation (orange triangle); (**b**) CS nanoparticles without incubation (purple diamond), one minute of incubation (blue square), and ten minutes of incubation (orange triangle).

Next, a quantitative evaluation was conducted by analyzing the nanoparticle concentration in the elution buffer and determining the nanoparticle mass retained on the mucosal surface and its dependence on the wash volume. The effect of different incubation times on the retention was also examined (Figure 5). When ACS nanoparticles were incubated for 1 min and washed at a flow rate of 2 mL/min, more particles were retained compared to the same experiment with no incubation stage (Figure 5a). After 10 min of incubation, nanoparticles were strongly bound to the mucosal membrane, and the retention percentages were higher than for 1 min incubation. The same trend was observed for CS nanoparticles (Figure 5b).

**Figure 6:**
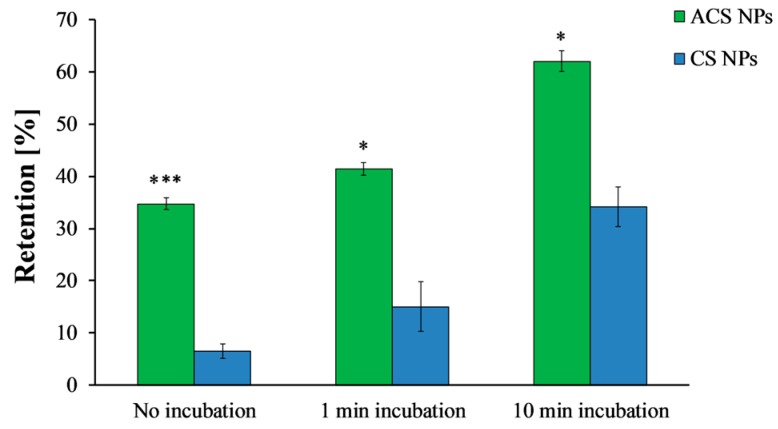
Final retention percentages of ACS nanoparticles (green) and CS nanoparticles (blue) obtained after different incubation time intervals (ANOVA, *p* < 0.0001, *n* = 3, *p* < 0.05, *n* = 3, respectively). (*) refers to statistically significant difference (*p* < 0.05), and (***) refers to statistically significant difference (*p* < 0.001).

The washing curves presented in Figure 5 were used to determine the retention at steady state, defined here as 95% of the final retention at the end of the experiment. Without incubation, ACS nanoparticles were initially washed away from the surface, reaching a steady state of 38% retention after 1.65 mL of wash volume (Figure 5a). CS nanoparticles, on the other hand, reached steady state at a lower value of 11% retention after 1.67 mL (Figure 5b). When the washing started after 1 min of incubation, a steady state of 44% retention was observed after 1.91 mL of wash volume for ACS nanoparticles, while CS nanoparticles reached a steady state of 19% retention after washing with 1.83 mL. Finally, after 10 min of incubation, the retention percentage of ACS nanoparticles leveled off to 64% after 2.17 mL of elution buffer, compared to 37% retention after washing with 2.90 mL for CS nanoparticles under the same conditions. These results indicate that the washing volume required to reach steady state is similar for both nanoparticle formulations, suggesting no great difference in the kinetics of reaching equilibrium. However, the steady state retention is lower for CS nanoparticles, indicating its lower mucoadhesion. Furthermore, the retention percentage at the end of the experiment, obtained after approximately 4 mL wash volume (Figure 6), demonstrates the differences in binding strength as well. A statistical analysis revealed that the retention values of both ACS and CS nanoparticles depends significantly on the incubation time (ANOVA, *p* < 0.0001, *n* = 3, *p* < 0.05, *n* = 3, respectively). The retention values of ACS nanoparticles at the end of the experiment were significantly higher than those of CS nanoparticles for all the examined incubation times (*t*-test, no incubation *p* < 0.001, 1 min incubation *p* < 0.05, 10 min incubation *p* < 0.05, *n* = 3).

The use of several theories of mucoadhesion can provide an explanation to the effect described. According to the diffusion theory of mucoadhesion, a liquid formulation interpenetrates via diffusion to the mucus layer. The interpenetration length depends directly on the contact time; as the contact time increases, so does the length [1]. The results displayed in Figure 5 and Figure 6 imply that diffusion processes are involved in the studied system since the mucoadhesion increases with incubation time (i.e., contact time). Furthermore, the adsorption theory of mucoadhesion suggests that after an initial contact, the molecules form secondary bonds such as hydrogen bonds and Van der Waals forces across the interphase [42]. Chemisorption, which is a subsection of the adsorption theory, assumes that the interactions across the interface are related to strong covalent bonds [1].

**Figure 7:**
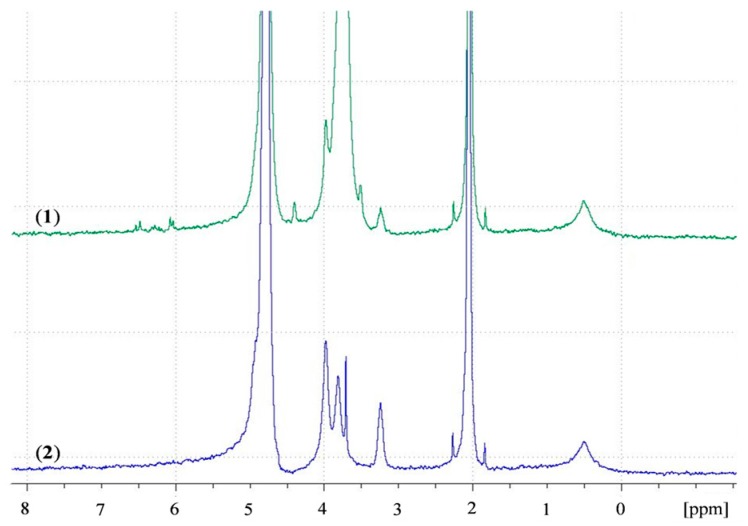
^1^H NMR spectra of (**1**) ACS nanoparticles; (**2**) CS nanoparticles in D_2_O.

A recent study by Shitrit and Bianco-Peled [16] found that acrylated CS presented improved mucoadhesion compared to unmodified CS. This improvement was attributed to the free acrylate group carried by PEGDA, associating with thiol end groups in mucin glycoprotein by a Michael-type addition reaction and form covalent bonds. To verify the presence of free acrylate end groups on the surface of ACS nanoparticles, we analyzed lyophilized CS and ACS nanoparticles, which were redispersed in D_2_O, using NMR spectra. Figure 7 presents the chitosan spectrum (in blue) with peaks in the range of δ = 3 to δ = 4 ppm. The ACS nanoparticle spectrum (in green) contains new types of protons at δ = 3.59–4.06 ppm, representing the two methylene groups in PEG repeating units. The peaks at δ = 6–6.7 ppm represent the vinyl end group protons [43], thus verifying the existence of free acrylate groups. The peaks located at δ = 4.87 ppm and at δ = 2.13 ppm are assigned to water protons and acetic acid, respectively. The primary amine group in the CS nanoparticle spectrum is represented by the peak located at δ = 3.3 ppm. The appearance of this peak implies that even after crosslinking with TPP, some free amine groups remain. The same peak is observed in the ACS nanoparticle spectrum with a decrease in signal. Similar results were obtained in previous studies on ACS [16,44]. These results support our hypothesis that free acrylate end groups on ACS nanoparticles may associate covalently with thiol groups present on mucin. These nanoparticles also interact strongly with the mucus layer as a result of entanglements and hydrogen bonds between long PEGDA chains and mucin glycoprotein. CS nanoparticles, on the other hand, have fewer possibilities for entanglements, and can only interact with mucin via hydrogen bonding or electrostatic interactions [5]. It is noted that the kinetics of Michael-type addition between thiol and acrylate groups is in the order of minutes. A recent study by Eshel-Green et al. found that thiol-acrylate bonds in PEG-based hydrogels are formed after one min of mixing at room temperature [27]. Another study that used thiolated CS mixed with PEGDA reported a gelation time of 25 min at 37 °C [45]. These studies suggest that the reaction between thiol and acrylate starts immediately upon mixing but reaches equilibrium in a matter of minutes.

#### 3.5.2. Nanoparticle-Mucin Aggregation

When a suspension of mucin is mixed with a solution of mucoadhesive polymer, the two components may aggregate due to covalent or electrostatic binding between the mucin and the polymer, supported by other forces such as hydrogen bonds and hydrophobic associations [46]. Similar association events occur between nanoparticles and mucin. Therefore, mucoadhesiveness can be evaluated by detecting the particle’s diameter in the presence of soluble mucin [20]. The mucoadhesive index (MI) value is calculated by dividing the diameter of the aggregates formed after incubation with mucin by the initial diameter of the particle.

The use of dilute polymer solutions was proposed in previous studies of polysaccharide-polysaccharide interactions, because it renders the effects of polymer exclusion negligible [46]. For this reason, we examined the association between mucin glycoprotein and the nanoparticles in dilute solutions. A soluble mucin fraction was separated from porcine stomach mucin in order to reduce the effects of mucin gelation and self-aggregation [47].

**Figure 8:**
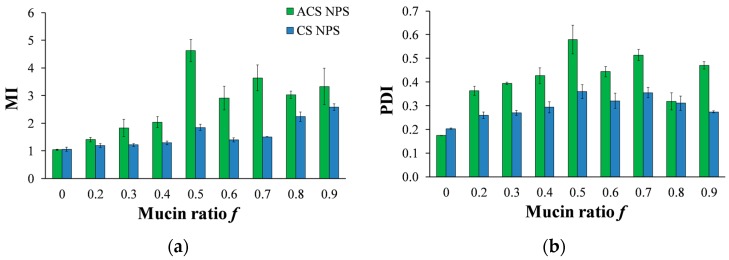
(**a**) MI values calculated for various compositions of mucin-ACS nanoparticle complexes (green) and mucin-CS nanoparticle complexes (blue) as measured by dynamic light scattering (DLS). (**b**) PDI for various compositions of mucin-ACS nanoparticle complexes (green) and mucin-CS nanoparticle complexes (blue) as measured by DLS.

A solution of nanoparticles with a fixed volume and concentration was incubated with soluble mucin solutions at increasing mucin ratio *f* (Equation (3)). Data in Figure 8a present the MI values against the mucin ratio *f*. It is noted that preliminary experiments with an incubation time of 5 h, as specified in a previous work [20], resulted in the same final diameter as incubation for just one min. Figure 8a demonstrates that in most of the mucin ratio range, MI values of ACS nanoparticle-mucin mixtures were higher than those of CS nanoparticles and mucin. If we consider MI values to be an indication of mucoadhesion strength, we can deduce that ACS nanoparticles are more mucoadhesive than CS nanoparticles.

The mucoadhesive strength (and thus the aggregate diameter) changes for different nanoparticle-mucin weight ratios (Figure 8a). For both ACS and CS nanoparticles, the MI ratio is significantly dependent on the mucin ratio *f* (ANOVA, *p* < 0.00001). For ACS nanoparticle-mucin mixtures in the range of *f* < 0.5, particle size increases with an increase in mucin fraction, from 152 ± 5 nm without mucin to 701 ± 0.4 at *f* = 0.5. When mucin is in excess, beyond the stoichiometric point, the MI values for ACS nanoparticles decrease from 4.9 ± 0.8 to 2.9 ± 0.4 at *f* = 0.9. A *t*-test for every two adjacent MI values confirmed the existence of a maximum in the MI vs. *f* curve (*p* < 0.05, *n* = 3), with the highest MI value obtained at the stoichiometric point (*f* = 0.5). As a control experiment, soluble mucin solutions were examined by DLS after dilution with buffer that does not contain nanoparticles. The results showed that the soluble mucin itself does not form measurable particles upon dilution. A similar test for CS nanoparticle-mucin mixtures did not detect a maximum in the MI vs. *f* curve: the MI values of CS nanoparticles grow with an increase in mucin fraction, from 1.0 ± 0.01 without mucin to 1.7 ± 0.14 at *f* = 0.5. MI values continue to rise when mucin is in excess, to 2.7 ± 0.11 at *f* = 0.9. 

**Figure 9:**
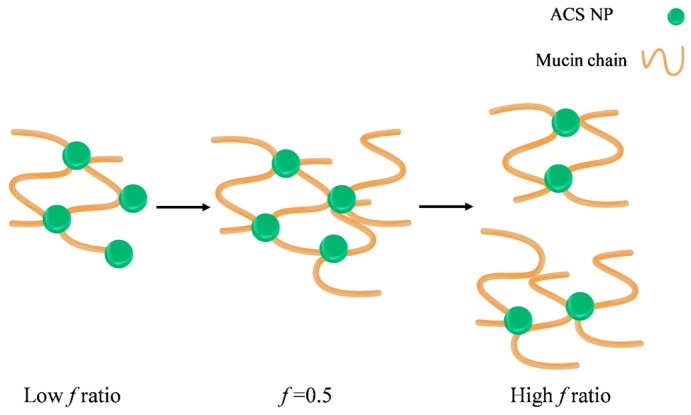
Schematic illustration of the proposed mechanism leading to the observed maximal complex diameter.

It was previously established by other methods that the binding between chitosan and mucin depends on the polymer to mucin ratio. Menchicchi et al. used an indirect colorimetric method to detect the amount of chitosan and mucin remaining in the solution after separating the polymer-mucin aggregates [46]. They found that the amount of mucin participating in the complex formation increased with the increase of polymer mucin ratio, up to a maximum value observed at the stoichiometric point. A similar effect was observed by Rossi et al., who calculated the rheological synergism parameter of polymer-mucin mixtures [48]. This study found a minimum value observed for 1:1 polymer to mucin weight ratio and suggested that the stoichiometry between chitosan hydrochloride and mucin is close to 1:1. To the best of our knowledge, this phenomenon is still unexplained, despite previous descriptions of an optimum in mucoadhesion for specific mucin-polymer ratios. We propose a possible mechanism to explain the complex formation and the observed optimal behavior (Figure 9).

In our experiment, the particle concentration is constant, while the mucin concentration increases. In this case, the nanoparticles act as contact points between the mucin chains, leading to the complex formation (Figure 9). As the mucin ratio increases, the number of mucin chains sequestered into the complex increases, until the size of the aggregate reaches its critical value. Then, the aggregates disassociate into smaller complexes, in order to maximize the interactions between the nanoparticles and the mucin chains. In addition, large aggregates have more repulsion interactions between negatively charged mucin chains, destabilizing the complex and causing it to break under stirring. In light of the results in this experiment, it is possible that the decrease in the complex size might not indicate a decrease in mucoadhesion. Instead, at high *f* ratio, the large excess of mucin chains reorganizes between the nanoparticles in a specific arrangement to make the most of the interactions. CS nanoparticle-mucin complexes are originally smaller than ACS nanoparticle-mucin aggregates. Therefore, it is possible that they do not reach a critical size, and so do not disassociate. 

To further support our results, we analyzed the PDI measured by DLS at increasing mucin ratio *f* (Figure 8b). As discussed above, the PDI value is a dimensionless measure of the broadness of the size distribution calculated from the cumulants analysis [32]. PDI values close to 1 indicate that the distribution is polydisperse. We hypothesize that the aggregation process of soluble mucin molecules with polymeric nanoparticles is likely to increase the polydispersity in solution. The results displayed in Figure 8b clearly show that the PDI values of mixtures of CS nanoparticles and soluble mucin are lower than those of ACS nanoparticles in the presence of mucin. The same maximum at *f* = 0.5 is observed in Figure 8a for mixtures of ACS nanoparticles and soluble mucin; however, this maximum is not statistically significant. These findings support the hypothesis that ACS nanoparticles have a greater tendency to associate with mucin than CS nanoparticles. 

## 4. Conclusions

We demonstrate that ACS can be crosslinked with TPP to form a novel nanoparticulate system. These nanoparticles were characterized and compared to CS nanoparticles. The size of the CS nanoparticles was found to depend significantly on the polymer to crosslinker ratio. In contrast, ACS particles obtained at different polymer/TPP ratios had almost the same size. The stability of the ACS nanoparticles was evaluated over a period of 30 days and was found to be better than that of the CS nanoparticles, with a diameter increase of only 12% compared to 23% for the CS nanoparticles. The nanoparticle yield was measured using two cycles of slow freezing and thawing. The yield was similar for ACS and CS.

The nanoparticle retention on a mucosal surface increased with the increase in incubation time for both formulations, which is in line with the prediction of the diffusion and the adsorption theories of mucoadhesion. The ACS nanoparticles presented higher retention on the mucosal tissue for all examined incubation times. These results imply that ACS nanoparticles can form more entanglements, thanks to the bulky PEGDA chain, as well as covalent and hydrogen bonds with mucins. The mucin particle method was utilized to study the aggregation of nanoparticles in the presence of mucin. These measurements demonstrated that the size of the aggregates increased with the increase in the ratio of mucin to CS nanoparticles. In ACS nanoparticle-mucin mixtures, a peak was observed in the curve of aggregate size vs. mucin ratio. This observation was attributed to the breakage of aggregates larger than a critical value.

## Figures and Tables

**Table 1:** The effect of chitosan (CS) and tripolyphosphate (TPP) concentration on the nanoparticle size, at a constant CS to TPP mass ratio of 8:1.

CS Concentration (mg/mL)	TPP Concentration (mg/mL)	Size by Intensity (nm)
2	1	354.6 ± 4.5
2	0.5	317.0 ± 6.6
2	0.25	249.0 ± 3.4
1	0.5	228.6 ± 0.8
1	0.25	209.4 ± 3.0

## Supplementary Materials

The following are available online at www.mdpi.com/2073-4360/10/2/106/s1, Figure S1: The molecular structure of ACS, Figure S2: ^1^H NMR spectra of (1) ACS, (2) PEGDA and (3) CS in D2O.
