# Supplementary material for: Acrylated Chitosan Nanoparticles with Enhanced Mucoadhesion
Source: Polymers (Basel). 2018 Jan 23;10(2):106. doi: 10.3390/polym10020106 (PMC6415080; doi:10.3390/polym10020106)
Supplement: Supplementary file 1 [file polymers-10-00106-s001.pdf]

Supplementary Material

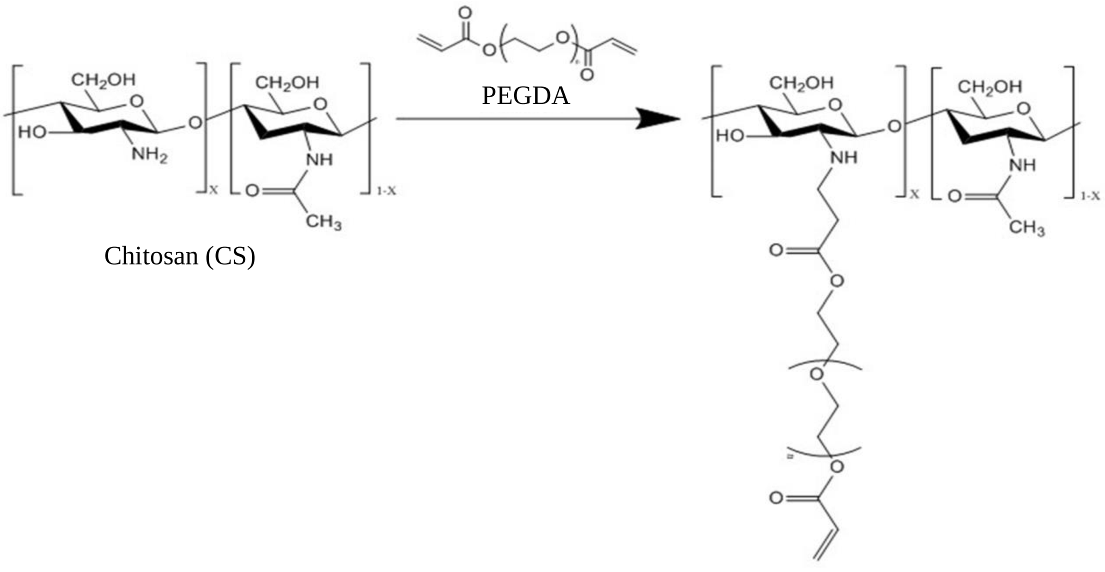

Figure S1. The molecular structure of ACS.

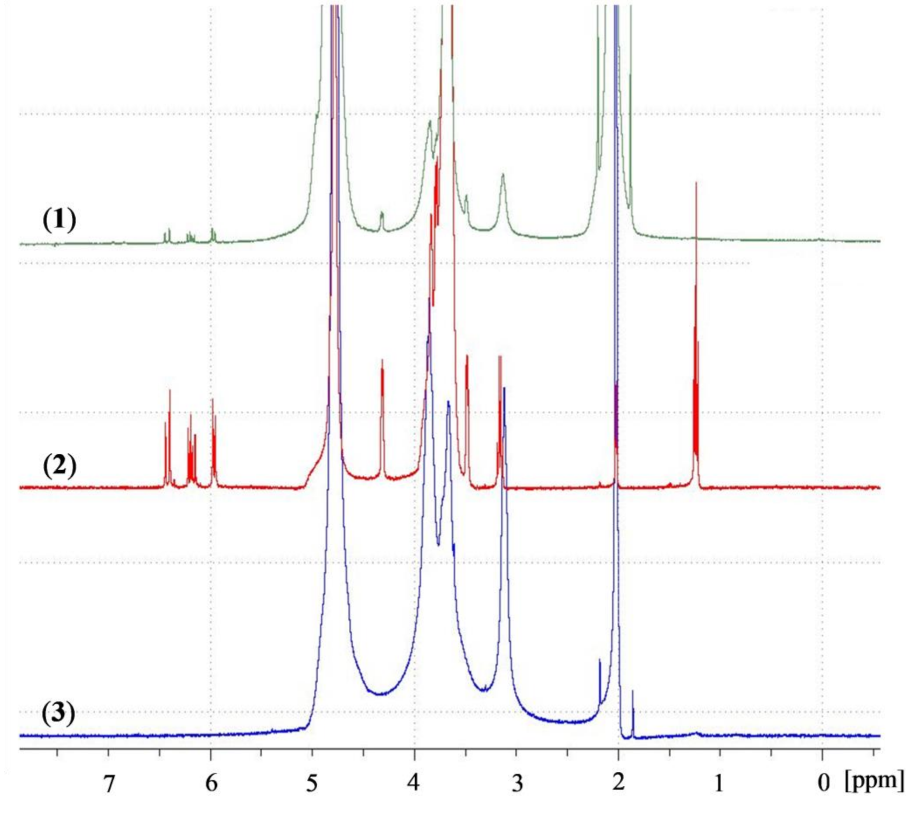

Figure S2. ^{1}H NMR spectra of (1) ACS, (2) PEGDA and (3) CS in D_2O.
